# Human epididymis protein 4, a novel potential biomarker for diagnostic and prognosis monitoring of lung cancer

**DOI:** 10.1111/crj.13774

**Published:** 2024-05-14

**Authors:** Tingting Zhang, Lanhe Chu, Wenchong Tan, Cuiping Ye, Hangming Dong

**Affiliations:** ^1^ Infection Management Department of Zengcheng Campus Nanfang Hospital, Southern Medical University Guangzhou China; ^2^ Department of Respiratory and Critical Care Medicine Nanfang Hospital, Southern Medical University Guangzhou China; ^3^ Department of Teaching and Research The Tenth Affiliated Hospital, Southern Medical University Dongguan China

**Keywords:** diagnosis, human epididymis protein 4, lung adenocarcinoma, lung cancer, prognosis

## Abstract

**Objective:**

This study aimed to explore the application value of human epididymis protein 4 (HE4) in diagnosing and monitoring the prognosis of lung cancer.

**Methods:**

First, TCGA (The Cancer Genome Atlas) databases were used to analyze whey‐acidic‐protein 4‐disulfide bond core domain 2 (WFDC2) gene expression levels in lung cancer tissues. Then, a total of 160 individuals were enrolled, categorized into three groups: the lung cancer group (*n* = 80), the benign lesions group (*n* = 40), and the healthy controls group (*n* = 40). Serum HE4 levels and other biomarkers were quantified using an electro‐chemiluminescent immunoassay. Additionally, the expression of HE4 in tissues was analyzed through immunohistochemistry (IHC). In vitro cultures of human airway epithelial (human bronchial epithelial [HBE]) cells and various lung cancer cell lines (SPC/PC9/A594/H520) were utilized to detect HE4 levels via western blot (WB).

**Results:**

Analysis of the TCGA and UALCAN (The University of Alabama at Birmingham Cancer Data Analysis Portal) databases showed that WFDC2 gene expression levels were upregulated in lung cancer tissues (*p* < 0.01). Compared with the control group and the benign group, HE4 was significantly higher in the serum of patients with lung cancer (*p* < 0.001). Receiver operating characteristic (ROC) analysis confirmed that HE4 had better diagnostic efficacy than classical markers in the differential diagnosis of lung cancer and benign lesions and had the highest diagnostic value in lung adenocarcinoma (area under the ROC curve [AUC] = 0.826). HE4 increased in early lung cancer and positively correlated with poor prognosis (*p* < 0.001). Moreover, the results of WB and IHC revealed that the expression of HE4 was increased in lung cancer cells (SPC/A549/H520) and lung cancer tissues but decreased in PC9 cells with a lack of exon EGFR19 (*p* < 0.05).

**Conclusion:**

Serum HE4 emerges as a promising novel biomarker for the diagnosis and prognosis assessment of lung cancer.

## Graphical abstract

Our study first used large public databases to analyze the expression of the WFDC2 gene in lung cancer tissues and then compared HE4 with classical tumor markers at the serological level. Finally, western blot and immunohistochemistry were used to detect the expression characteristics of HE4 in lung cancer cells and tissues. Our results show that serum HE4 emerges as a promising novel biomarker for the diagnosis and prognosis assessment of lung cancer.

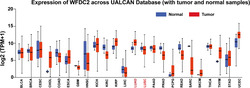

## 1 INTRODUCTION

According to the Global Cancer Statistics 2020, lung cancer is the second most commonly diagnosed cancer and remains the leading cause of cancer death, with an estimated 2.2 million new cancer cases and 1.8 million deaths.^1^ Lung cancer poses a substantial threat to human health, primarily due to its association with metastasis and recurrence. The early stages of lung cancer often present with subtle clinical symptoms, leading to misdiagnosis as benign conditions. Consequently, many patients are diagnosed with intermediate‐to‐advanced‐stage disease, missing the optimal window for surgical intervention. Currently, three main screening methods for lung cancer exist, including imaging examinations, cytology or histology techniques, and biomarkers. Among these, serum biomarkers are crucial in clinical diagnosis and treatment, given their non‐invasive and non‐radiative nature. Presently recommended biomarkers for lung cancer include carcinoembryonic antigen (CEA), progastrin‐releasing peptide (ProGRP), cytokeratin 19 fragment (Cyfra21‐1), neuron‐specific enolase (NSE), and squamous cell carcinoma antigen (SCC). However, these markers exhibit certain limitations in sensitivity and specificity for aiding lung cancer diagnosis.^2^, ^3^ Consequently, there is an urgent need to explore novel biomarkers with better diagnostic efficiency that can provide prognostic information and guide immunotherapy, which would significantly impact identifying individuals who would benefit from early screening and intervention.

Human epididymis protein 4 (HE4), a small secretory protein, is the product of the whey‐acidic‐protein 4‐disulfide bond core domain 2 (WFDC2) gene. WFDC2 belongs to the protease inhibitor family, contributing to protective immunity.^4^, ^5^ While HE4 has been extensively studied as a biomarker for ovarian cancer,^6^, ^7^, ^8^ researchers such as Bingle et al. have shown its expression in various locations, including the oral cavity, nasopharynx, respiratory tract, salivary glands, and lungs, highlighting its broad tissue distribution in 2006.^9^ Also, a human genome‐wide gene expression microarray showed that 231 differentially expressed genes (DEGs) changed in response to HE4, involving the mitogen‐activated protein kinase (MAPK) signal, extracellular matrix (ECM) receptor, cell cycle, and steroid biosynthesis pathway.^10^ Moreover, animal experiments demonstrated that the knockout of the WFDC2 gene in mice resulted in severe dyspnea and type I alveolar cell death, suggesting the involvement of WFDC2 in lung function expression.^11^ The above results indicate that overexpression of HE4 plays an important role in tumor progression and is associated with lung cancer. But up to now, research on HE4 in lung cancer has predominantly focused on serological detection,^12^, ^13^, ^14^, ^15^ lacking basic experimental demonstration, and its biological function remains poorly understood. The relationship between HE4 expression and the diagnosis and progression of lung cancer remains unclear.

Therefore, our study first used large public databases to analyze the expression of the WFDC2 gene in lung cancer tissues and then compared HE4 with classical tumor markers at the serological level. Finally, western blot (WB) and immunohistochemistry (IHC) were used to detect the expression characteristics of HE4 in lung cancer cells and tissues. Furthermore, a more comprehensive evaluation was conducted on the application value of HE4 in the diagnosis and prognosis of lung cancer.

## 2 MATERIALS AND METHODS

### 2.1 Biological database analysis

For the analysis, publicly available databases, including The Cancer Genome Atlas (TCGA: http://www.cancer.gov/) and The University of Alabama at Birmingham Cancer Data Analysis Portal (UALCAN: http://ualcan.path.uab.edu/analysis.html), were utilized. The search criteria were restricted to “lung cancer” cases, and efforts were made to exclude duplicate data and eliminate entries lacking clinical information. Under these specified conditions, the expression of the “WFDC2” gene was assessed using the respective functionalities provided by these databases.

### 2.2 Patients and sample collection

#### 2.2.1 Participant characteristics

This study included 160 patients diagnosed at the *Nanfang Hospital Zengcheng Campus, Southern Medical University*, between January 2020 and August 2023. Three distinct cohorts were established based on diagnostic criteria: the lung cancer cohort (*n* = 80), the benign lesions cohort (*n* = 40), and the healthy controls cohort (*n* = 40). Table 1 provides the basic information for individuals in each group. Simultaneously, cancer tissues and adjacent normal tissues were obtained from eight early‐stage lung cancer patients post‐surgery, sourced from the *Pathology Department* at *Nanfang Hospital, Southern Medical University*.

**TABLE 1:** Basic clinical characteristics of enrolled patients.

Information	Lung cancer		Benign lesion		Healthy control		*p*‐value
	No.	%	No.	%	No.	%	
Number	80	50.0	40	25.0	40	25.0	
Gender							0.131
Male	56	70.0	28	70.0	21	52.5	
Female	24	30.0	12	30.0	19	47.5	
Age (years)							0.216
≥65	41	51.25	15	37.5	15	37.5	
<65	39	48.75	25	62.5	25	62.5	
Smoking history							0.117
Yes	50	62.5	19	47.5	/	/	
No	30	37.5	21	52.5	/	/	
Pathologic types							
LUAD	50	62.5					
LUSC	18	22.5					
SCLC	12	15.0					
TNM stages							
Stages I–II	25	31.35					
Stages III–IV	55	68.75					
Treatment status							
No treatment	30	37.50					
Treatment	50	62.50					
Clinical response							
CR	10	20.0					
PR	10	20.0					
SD	14	28.0					
PD	16	32.0					

Abbreviations: CR, complete response; LUAD, lung adenocarcinoma; LUSC, lung squamous cell carcinoma; PD, progressive disease; PR, partial response; SCLC, small cell lung cancer; SD, stable disease; TNM, tumor, node, and metastasis.

#### 2.2.2 Inclusion and exclusion criteria

The inclusion criteria were as follows: (1) lung cancer patients adherence to the Chinese Medical Association's guidelines for Clinical Diagnosis and Treatment of Lung Cancer (2023 version),^16^ normal creatinine levels (male < 115 μmol/L, female < 80 μmol/L), and availability of pathological and imaging data for staging and solid tumor response assessment. Solid tumor response was categorized according to RECIST 1.1 criteria as complete response (CR), partial response (PR), stable disease (SD), or progressive disease (PD); (2) the benign group includes patients with benign lung lesions diagnosed by clinicians, such as pneumonia, lung mass, chronic obstructive pulmonary disease, and so forth; (3) the healthy control group came from the physical examination population, which had no underlying disease and no history of medication in the past 1 month. The exclusion criteria encompassed patients with other malignancies or severe underlying diseases, such as severe infections or advanced liver and kidney disorders.

### 2.3 Serum biomarker measurements

Serum samples were collected using coagulated tubes, separated by centrifugation at 2136 *g* for 15 min, transferred to new Eppendorf tubes, and stored at −80°C until analysis. The serum levels of HE4, ProGRP, CEA, NSE, Cyfra21‐1, and SCC were determined using the electro‐chemiluminescent immunoassay method with the Cobas e602 system from Roche, along with its corresponding kit. The experiments adhered to the instrument operating procedures and laboratory Standard Operating Procedures (SOP) standards. The accuracy of the test results was maintained through instrument quality control and calibration processes. These measures ensured the reliability and precision of the obtained data.

### 2.4 Cell culture

Human hepatoma cell lines (HepG2, Huh7, MHCC97H), normal liver cell lines (LO2), normal esophageal epithelial cell lines (Het‐1A), and esophageal cancer (EC) cell lines (KYSE‐150, Eca‐109) were generously provided by the Department of Occupational Health and Medicine at the School of Public Health, Southern Medical University (Guangzhou, China). Lung cancer cell lines (SPC, PC9, A594, and H520) were purchased from ATCC. To ensure their integrity, all cell lines underwent validation for the absence of mycoplasma contamination using the MycAway™‐Color One‐Step Mycoplasma Detection Kit (Yeasen, Shanghai, China). The cell lines LO2, HepG2, Huh7, MHCC97H, Het‐1A, KYSE‐150, and Eca‐109 were cultured in Dulbecco's modified Eagle's medium (DMEM; Gibco, USA), while human bronchial epithelial (HBE) cells were cultured in keratinocyte medium (KM; ScienCell). SPC, PC9, A594, and H520 cells were incubated in a 1640 medium (ThermoFisher, China). All media were supplemented with 10% fetal bovine serum (FBS; Gibco) and 1% streptomycin and penicillin, and cells were maintained in a 5% CO_2_ atmosphere at 37°C.

### 2.5 Western blot

The cell lysis process involved a lysis buffer containing a protease inhibitor, a phosphatase inhibitor, and phenylmethylsulfonyl fluoride (PMSF). Following lysis, the cell lysate underwent centrifugation for 15 min at 12 000 *g* at 4°C. The resulting supernatant was utilized for WB analysis. The protein fraction obtained was boiled and separated via electrophoresis on 12% or 15% sodium dodecyl sulfate (SDS)‐polyacrylamide gels. Subsequently, proteins were detected by immunoblotting using the LI‐COR Odyssey infrared imaging system (LICOR Bioscience). Band intensities were quantified using Image J software. The antibodies employed in this analysis included HE4 (Santa Cruz, USA, sc‐293 473), glyceraldehyde 3‐phosphate dehydrogenase (GAPDH) (CST, USA, #5174), and ACTIN (Proteintech, China, 66009‐1‐Ig).

### 2.6 Immunohistochemistry

IHC was performed on paraffin sections of tumor tissues obtained from lung cancer patients using the standard LSAB protocol (Dako, USA). The primary antibody against HE4 (dilution 1:50) was employed, with species‐matched IgG as a negative control. Positive results were indicated by brown staining, and the intensity of the IHC results was quantified using Image Pro Plus 6.0, with average optical density (AOD) used as the measure for analysis.

### 2.7 Statistical analyses

The chi‐square (*χ* ^2^) test was employed for comparing categorical variables. The distribution of experimental data was assessed using the Shapiro–Wilk test. Two‐tailed unpaired Student's *t*‐test or one‐way analysis of variance (ANOVA) was utilized for normally distributed data, and the results were presented as mean ± standard deviation (SD). Non‐normally distributed data were compared using the Mann–Whitney *U* test, and the outcomes were described as median (range). The Kruskal–Wallis test was applied to compare three groups with Bonferroni‐corrected significant values. Receiver operating characteristic (ROC) curve analysis was performed to evaluate the predictive abilities of biomarkers, and the cut‐off value, corresponding sensitivity, and specificity were determined using the Youden index. All statistical analyses were conducted using SPSS software Version 26.0 (IBM, USA), and statistical significance was set at *p* < 0.05.

## 3 RESULTS

### 3.1 The expression of the WFDC2 gene in lung cancer tissues

**FIGURE 1:**
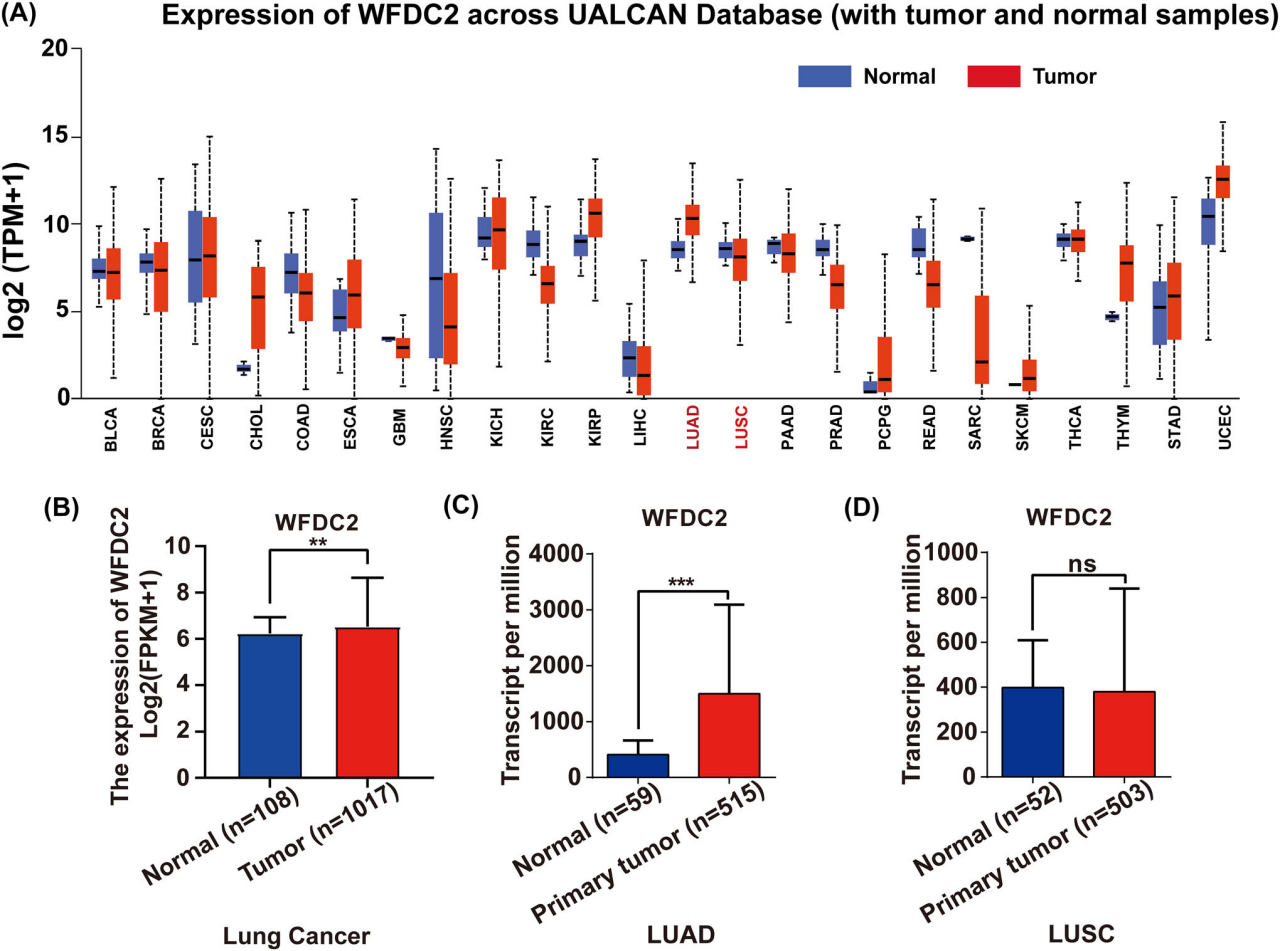
The expression of the WFDC2 gene in lung cancer tissues. (A) Expression of WFDC2 in various tumor tissues. (B–D) Expression of WFDC2 in lung cancer, LUAD, and LUSC, respectively. **p < 0.01; ***p < 0.001; ns, no significance.

The analysis of WFDC2 expression in different tumors using the UALCAN database revealed pan‐cancer results indicating expression of WFDC2 mRNA levels in a variety of tumor tissues (Figure 1A). To further explore the expression patterns, specifically in lung cancer, mRNA expression data from TCGA were analyzed. The results demonstrated increased mRNA levels of WFDC2 in both overall lung cancer and lung adenocarcinoma (LUAD) (Figure 1B,C), with no significant difference observed in lung squamous cell carcinoma (LUSC) (Figure 1D).

### 3.2 Comparison of lung cancer‐related tumor marker levels in serum

To verify the above hypothesis, we conducted electro‐chemiluminescent immunoassays to measure the concentrations of HE4 and five classical lung cancer‐related tumor markers, including CEA, ProGRP, Cyfra21‐1, NSE, and SCC (Table 2). The results revealed a significant increase in HE4 concentration in lung cancer patients compared with benign lesions and healthy controls (*p* < 0.001). Additionally, serum levels of HE4, Cyfra21‐1, and CEA exhibited a notable increase in stages III–IV compared with stages I–II (*p* < 0.001). Based on these results, we suggest that HE4 could serve as a novel and potentially significant biomarker, playing a major role in distinguishing and diagnosing lung cancer.

**TABLE 2:** The median value of serum HE4 and classical biomarkers in different groups.

Diagnosis	No.	HE4 (pmol/L)	ProGRP (pg/mL)	SCC (ng/mL)	NSE (ng/mL)	Cyfra21‐1 (μg/L)	CEA (ng/mL)
		Median (range)	Median (range)	Median (range)	Median (range)	Median (range)	Median (range)
Healthy control^a^	40	53.84 (36.74–83.76)	40.68 (23.87–69.58)	1.02 (0.50–0.91)	11.39 (4.74–17.23)	1.88 (1.10–3.50)	1.90 (0.51–4.88)
Benign lesion^b^	40	64.11 (34.15–117.00)	46.30 (22.33–100.60)	0.88 (0.48–11.24)	11.45 (7.56–17.90)	1.91 (0.79–5.68)	2.22 (0.65–9.61)
Lung cancer	80	101.75 (38.78–703.50)	47.49 (17.01–5000.00)	1.32 (0.38–26.86)	14.10 (6.11–196.70)	3.94 (1.23–48.53)	3.53 (0.63–342.00)
LUAD	50	112.55 (42.32–703.50)	43.87 (17.01–147.60)	1.19 (0.40–26.86)	14.10 (7.77–180.40)	3.82 (1.49–48.53)	5.29 (0.63–342.00)
LUSC	18	87.99 (38.78–403.10)	52.68 (18.81–99.13)	1.67 (0.38–11.28)	13.83 (6.11–52.16)	4.96 (1.32–22.13)	2.48 (1.05–145.20)
SCLC	12	103.00 (44.70–236.70)	373.9 (49.97–5000.00)	1.37 (0.56–6.88)	21.94 (6.34–196.70)	3.45 (1.23–8.39)	3.82 (0.90–19.89)
TNM stages							
Stages I–II^c^	25	67.40 (38.78‐191.60)	44.99 (17.01–74.77)	1.33 (0.38–5.26)	12.69 (7.75–18.59)	2.13 (1.23–5.35)	1.48 (0.68–8.56)
Stages III–IV	55	152.20 (44.70–703.50)	50.39 (17.04–5000.00)	1.31 (0.40–26.86)	15.15 (6.11–196.70)	5.82 (1.32–48.53)	5.63 (0.63–342.00)

Abbreviations: CEA, carcinoembryonic antigen; Cyfra21‐1, cytokeratin 19 fragment; HE4, human epididymis protein 4; LUAD, lung adenocarcinoma; LUSC, lung squamous cell carcinoma; NSE, neuron‐specific enolase; ProGRP, progastrin‐releasing peptide; SCC, squamous cell carcinoma antigen; SCLC, small cell lung cancer; TNM, tumor, node, and metastasis.

^a^ Comparison between lung cancer and healthy control (HE4: *p* < 0.001, ProGRP: *p* = 0.007, SCC: *p* = 0.041, NSE: *p* = 0.001, Cyfra21‐1: *p* < 0.001, CEA: *p* < 0.001).

^b^ Comparison between lung cancer and benign lesion (HE4: *p* < 0.001, ProGRP: *p* = 0.947, SCC: *p* = 0.023, NSE: *p* = 0.001, Cyfra21‐1: *p* < 0.001, CEA: *p* = 0.001).

^c^ Comparison between stages I–II and III–IV (HE4: *p* < 0.001, ProGRP: *p* = 0.152, SCC: *p* = 0.767, NSE: *p* = 0.020, Cyfra21‐1: *p* < 0.001, CEA: *p* < 0.001).

### 3.3 Diagnostic performance of HE4 and classical biomarkers for different clinicopathological subtypes of lung cancer

**FIGURE 2:**
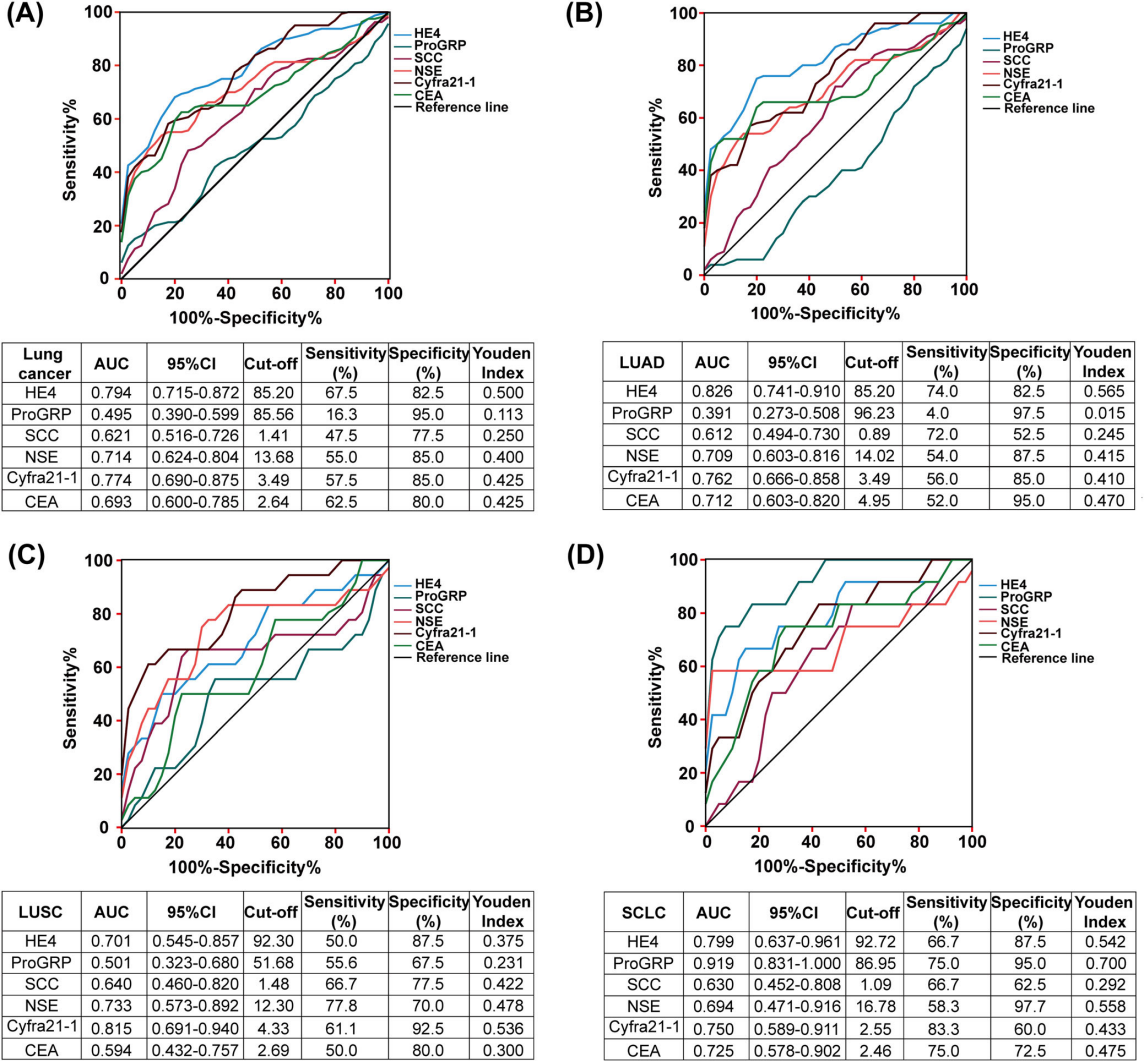
Diagnostic efficiency of HE4 and 5 classical tumor markers in lung cancer and its pathological subtypes. (A–D) ROC curve analysis of the diagnostic efficacy of HE4, ProGRP, SCC, NSE, Cyfra21‐1, and CEA tumor markers in detecting lung cancer (*n* = 80), LUAD (*n* = 50), LUSC (*n* = 18), and SCLC (*n* = 12), respectively.

We constructed ROC models for lung cancer patients and benign disease populations, and the corresponding parameters, including the area under the ROC curve (AUC), 95% confidence interval (CI), sensitivity, specificity, cut‐off value, and Youden index, are presented in Figure 2. The AUC values for determining lung cancer were HE4 (0.794) > Cyfra21‐1 (0.774) > NSE (0.714) > CEA (0.693) > ProGRP (0.495). With an optimal cut‐off value of 85.2 pmol/L, HE4 demonstrated a sensitivity of 67.5% and a specificity of 82.5%, yielding the highest Youden index of 0.5 among all tumor markers (Figure 2A). Similar trends were observed in LUAD, with an AUC value of 0.826, a sensitivity of 74.0%, a specificity of 82.5%, and a Youden index of 0.565, the highest among all markers (Figure 2B). However, for LUSC and small cell lung cancer (SCLC), Cyfra21‐1 (AUC: 0.815, sensitivity: 61.1%, specificity: 92.5%, Youden index: 0.536) and ProGRP (AUC: 0.919, sensitivity: 75.0%, specificity: 95.0%, Youden index: 0.700) were identified as the most suitable biomarkers, respectively (Figure 2C,D).

### 3.4 Analysis of related factors affecting HE4 levels

**FIGURE 3:**
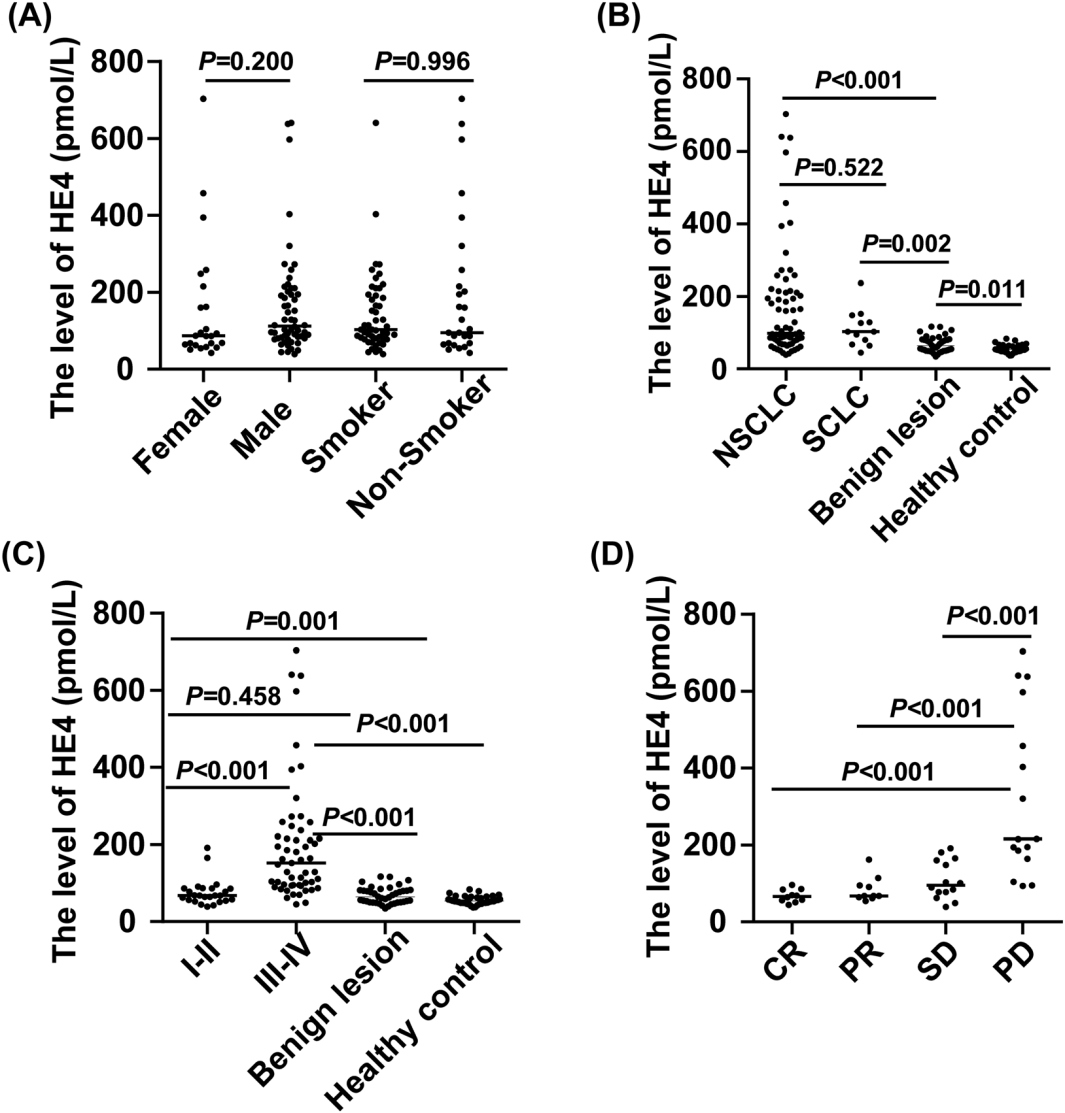
Analysis of related factors affecting HE4 levels, such as gender, smoking, subtype, stage, and disease progression. (A) Comparison of serum HE4 in gender and smoking patients. (B) Comparison of serum HE4 in NSCLC and SCLC. (C) Comparison of serum HE4 in stages I–II and III–IV. (D) Comparison of serum HE4 in the different prognoses of lung cancer patients.

To further establish serum HE4 as a suitable biomarker for diagnosing and predicting lung cancer, we analyze related factors that affect HE4 levels, such as gender, smoking, subtype, stage, and disease progression. Interestingly, there were no significant differences observed in serum HE4 levels between females and males or between smokers and non‐smokers (*p* = 0.200, *p* = 0.996; Figure 3A). Moreover, serum HE4 levels were elevated in both non‐small cell lung cancer (NSCLC) and SCLC compared with benign lesions or healthy controls, with no significant difference observed between NSCLC and SCLC (Figure 3B). Additionally, serum HE4 levels in lung cancer patients at stages I–II were significantly higher than those in healthy controls (*p* < 0.001), suggesting an elevation of HE4 in the early stages of lung cancer. Subsequently, a substantial increase in HE4 levels was observed in patients at stages III–IV as the disease progressed (Figure 3C). Furthermore, the association between HE4 and disease progression during therapy was evaluated. Higher levels of serum HE4 were associated with disease progression, as patients with PD exhibited significantly higher serum HE4 levels compared with patients in remission or with relatively stable conditions (*p* < 0.001; Figure 3D).

### 3.5 The diagnostic value of serum biomarkers for early lung cancer

To explore the potential of HE4 in diagnosing early stages of lung cancer, a subset of 25 patients at stages I–II was selected. The initial screening positive rates based on serum levels of HE4 were compared with those of other classical biomarkers. In this analysis, the positivity rate of HE4 reached 32%, surpassing that of Cyfra21‐1 (28%) and NSE (12%). Notably, ProGRP, SCC, and CEA positivity rates were all less than 10% (8%, 8%, and 4%, respectively) (Table 3).

**TABLE 3:** Comparison of the screening ability of biomarkers for early lung cancer.

Biomarkers	Number	Positive reaction	Negative reaction	Positive rate (%)
HE4	25	8	17	32
ProGRP	25	2	23	8
SCC	25	2	23	8
NSE	25	3	22	12
Cyfra21‐1	25	7	18	28
CEA	25	1	24	4

Abbreviations: CEA, carcinoembryonic antigen; Cyfra21‐1, cytokeratin 19 fragment; HE4, human epididymis protein 4; NSE, neuron‐specific enolase; ProGRP, progastrin‐releasing peptide; SCC, squamous cell carcinoma antigen.

### 3.6 Comparison of the diagnostic performance of multimarker tests in clinical analysis

Recognizing that classical tumor markers may not be the ideal diagnostic test for lung cancer detection, an innovative approach involving the combination of classical biomarkers with HE4 was explored to enhance sensitivity and accuracy in detection. In this experiment, ProGRP could distinguish between NSCLC and SCLC with an accuracy of 89.71% and 83.33%, respectively. Interestingly, when combined with HE4, the correct diagnostic rate for NSCLC increased to 91.18%, with little change in the correct rate for SCLC. Furthermore, the ProGRP, HE4, and NSE combination improved the overall accuracy to 96.25% (Table 4).

**TABLE 4:** Application of biomarker combinations in distinguishing NSCLC from SCLC.

Combination		NSCLC	SCLC	Percentage correct
ProGRP	NSCLC	61	7	89.71
	SCLC	2	10	83.33
		Overall percentage		88.75
ProGRP + HE4	NSCLC	62	6	91.18
	SCLC	2	10	83.33
		Overall percentage		90.00
ProGRP + HE4 + NSE	NSCLC	67	1	98.53
	SCLC	2	10	83.33
		Overall percentage		96.25

Abbreviations: HE4, human epididymis protein 4; NSCLC, non‐small cell lung cancer; NSE, neuron‐specific enolase; ProGRP, progastrin‐releasing peptide; SCLC, small cell lung cancer.

### 3.7 Expression of HE4 in lung cancer cells and tissues

**FIGURE 4:**
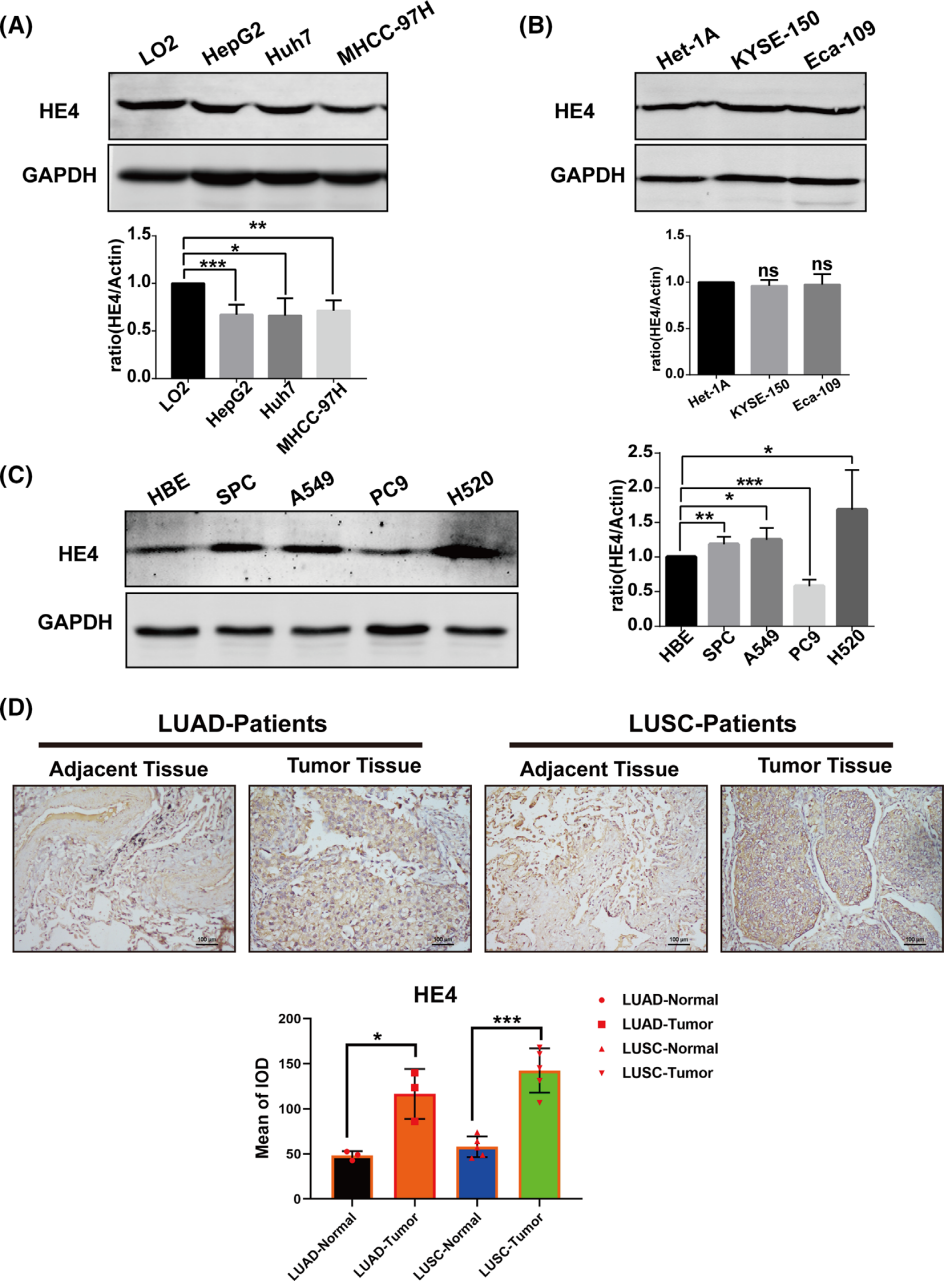
Expression of HE4 in hepatocellular carcinoma cells, esophageal cancer cells, lung cancer cells, and tissues. (A) The expression of HE4 was reduced in hepatocellular carcinoma cells. (B) The expression of HE4 in esophageal cancer cells showed no significant difference. (C) The expression of HE4 was upregulated in lung cancer cells (SPC/A549/H520) but downregulated in PC9 cells. (D) The expression of HE4 in lung cancer tissues was higher than that in adjacent tissues. *p < 0.05; **p < 0.01; ***p < 0.001; ns, no significance.

It had been found that HE4 could exert an important role in lung cancer; thus, we first investigated whether HE4 is specially expressed in lung cancer and other solid tumors. The protein levels of HE4 were further assessed in hepatocellular carcinoma (HCC) cells (HepG2/Huh7/MHCC97H; Figure 4A) and EC cells (KYSE‐150/Eca109; Figure 4B). Unexpectedly, HE4 expression was found to have no statistical significance in EC cells; however, it was downregulated in HCC cells. Notably, the expression of HE4 was significantly increased in lung cancer cells (SPC/A549/H520) but decreased in PC9 cells, possibly due to the loss of EGFR19 exons (E746‐A750) (Figure 4C). To further validate these findings, we used IHC analysis and found that the expression levels of HE4 in LUAD and LUSC tissues were higher than those in adjacent tissues (*p* < 0.05; Figure 4D).

## 4 DISCUSSION

It is well established that the survival time of lung cancer patients is closely linked to the timing of their clinical diagnosis. The 5‐year survival rate decreases with disease progression, ranging from 55.5% for stage I to a mere 5.3% for stage IV patients.^17^ In order to improve diagnostic sensitivity, low‐dose computed tomography (LDCT) scans are commonly used in lung cancer screening in numerous regions, exhibiting satisfactory performance. However, recent studies have indicated that LDCT is associated with a high false‐positive rate, risks of overdiagnosis, and cumulative radiation exposure. Consequently, it may not be optimal for lung cancer screening in low‐risk populations.^18^, ^19^ While there are still limitations in applying serum biomarkers for lung cancer diagnosis, their detection is simple, safe, low cost, and has a wide clinical audience. As an emerging tumor marker, HE4 has attracted much research attention since its discovery because it is a member of the WFDC family homologous to SLPI and Elafin proteins.^6^ Studies have shown that WFDC protein is widely expressed in the human epididymis and is associated with lung homeostasis and respiratory diseases.^20^ Previous studies have reported that HE4 is interconnected with a variety of immune genes and can regulate the immune defense function of the tumor body by participating in the regulation of B cells in the humoral immune microenvironment and the co‐expression of secretory leukocyte protease inhibitor genes.^5^ It can also promote angiogenesis and immunosuppress the tumor microenvironment by regulating signal transducer and activator of transcription 3 (STAT3) target genes.^21^ At the same time, related studies have found that the HE4‐encoding gene WFDC2 can induce the expression of matrix metalloproteinase 2 (MMP2) by activating the AKT signaling pathway, promote epithelial–mesenchymal transition, and then promote the metastasis of ovarian cancer cells.^22^ Knocking down WFDC2 can inhibit the proliferation, migration, and invasion of A549 cells.^23^ It has been confirmed that high HE4 expression affects the occurrence and development of tumors, but its biological behavior in lung cancer has been rarely reported, and its use as a lung cancer marker is still in the exploratory stage.

In this study, we first analyzed the TCGA database and determined that WFDC2 was overexpressed in lung cancer tissues, which suggested that WFDC2 may be an oncogene in lung cancer and participate in the regulation of related tumor mechanisms, and its gene product HE4 may play a role in disease monitoring. Then, we selected 160 samples and compared the expression of HE4 with ProGRP, Cyfra21‐1, NSE, SCC, and CEA in the three groups of people, confirming that HE4 has the characteristics of characterizing lung cancer. Through the analysis of the construction of the ROC model in this retrospective study, it was found that HE4 appears to be more accurate at predicting lung cancer than the other classical tumor markers, with higher sensitivity and specificity, which is consistent with the results reported in relevant domestic and foreign literature.^13^, ^14^, ^15^, ^24^ It was found that in LUAD, the diagnostic efficiency was highest among subtypes, which suggests that there was a correlation between HE4 expression and cell type. At the same time, we also observed that HE4 was significantly higher in the serum of NSCLC and SCLC patients than in the benign group. However, there was no statistical difference in the expression levels between the two, indicating that HE4 alone did not exhibit specificity towards the two major pathological subtypes, consistent with the findings reported by W. Liu et al.^25^ However, it is essential to note that our cohort study requires the enrollment of more patients for further follow‐up analysis. Furthermore, the combined application of HE4 with other biomarkers yielded varied effects. Some studies have reported that ProGRP is highly specific for SCLC.^26^ This study showed through retrospective case analysis that ProGRP alone can distinguish NSCLC from SCLC with an accuracy of 88.75%, while the combination of ProGRP + HE4 + NSE can accurately distinguish NSCLC from SCLC; the accuracy increased to 96.25%, suggesting that the synergistic effect of HE4 combined with other biomarkers enhances the identification of lung cancer subtypes. This finding may provide more reference for the application of clinical indicator combinations.

It has been previously suggested that HE4 is a potential marker for early‐stage lung cancer.^3^, ^14^, ^27^ While the serum levels of HE4 in patients at stages I–II were higher than those in healthy controls, its superiority over benign lesions was not evident. However, a retrospective analysis of HE4 and other biomarkers in patients with early lung cancer revealed a positivity rate of 32%, which still has certain advantages over classical biomarkers. Therefore, to a certain extent, detecting HE4 expression levels may help in the early diagnosis of lung cancer and help screen more beneficiary groups. Simultaneously, we observed a significant increase in HE4 levels in patients with advanced disease (stages III–IV), speculating that elevated HE4 expression may be an important feature of lung cancer progression. Scholars Sun and Mo have reported that lung cancer patients with high HE4 levels have significantly shorter survival times than those with lower levels.^28^, ^29^ We conducted a RECIST 1.1 assessment on the treated patients and found that the HE4 levels in the PD group were significantly higher than those of patients in the CR, PR, and SD groups, which confirmed our conjecture and also showed that HE4 can be used as a prognostic monitoring indicator for lung cancer.^28^ Furthermore, the serum levels of HE4 in the lung cancer cohort were not influenced by smoking or gender, aligning with the literature.^14^, ^15^, ^29^, ^30^ The reason may be that the impact of lung cancer progression on HE4 covers gender and subtle changes produced by smoking.

Combined with the pan‐cancer analysis results, we selected HCC cells with downregulated expression of WFDC2 and EC cells with upregulated expression for comparative experiments. Through WB detection technology, we found that the expression of HE4 was not increased in both malignant tumor cells. This finding further emphasizes that HE4 is not a universally expressed tumor protein across all cancer types and is consistent with its specificity as a biomarker. Importantly, HE4 levels were upregulated in lung cancer cells (SPC/A549/H520) but downregulated in PC9 cells. PC9 is a LUAD cell line with EGFR19 exon deficiency and sensitivity to EGFR‐TKI drugs such as osimertinib and ametinib. It has been reported that the expression of HE4 in ovarian cancer is related to epidermal growth factor activity and pointed out that HE4 has a potential interaction with epidermal growth factor receptor (EGFR) or other cell surface receptors.^31^ Related studies have found that knocking down the HE4 gene in lung cancer can lead to inactivation and downregulation of EGFR expression, thereby inhibiting EGFR downstream signaling pathways and the growth and metastasis of lung cancer cells.^32^, ^33^ Moreover, our experiments have found that PC9 cells downregulate EGFR expression and affect HE4 expression due to EGFR gene mutations. In this regard, we speculate that HE4 and EGFR have mutual regulatory effects at the transcription level or the protein level, but the regulatory mechanism still needs further study. Finally, we further confirmed the overexpression of HE4 in lung cancer tissues through IHC. The results showed that, unlike the expression trend of WFDC2 mRNA in the TCGA database, HE4 was highly expressed in both LUAD and LUSC tissues compared with adjacent tissues. This result confirmed the conclusion that HE4 was increased in serum and cell levels and strongly supported the idea of HE4 as a biomarker for lung cancer. Considering that gene transcription and translation is a complex process, however, there are few pathological samples enrolled in LUSC, so there may be a certain degree of bias, and further in‐depth research is needed in the future.

## 5 CONCLUSION

Our data suggest that serum HE4 is a better tumor biomarker for diagnosing and predicting lung cancer than other classical markers, particularly in LUAD. Combining HE4 with ProGRP and NSE could further enhance the ability to differentially diagnose NSCLC from SCLC. Although histopathology and imaging remain the gold standard methods for diagnosis, our study substantiates that HE4 would be a novel potential biomarker for diagnosing and predicting early‐stage lung cancer, which would offer a novel avenue for therapeutic targeting and present a strategic approach to improve the prognosis of lung cancer patients.

## AUTHOR CONTRIBUTIONS

**Tingting Zhang**: Conceptualization; formal analysis; writing—original draft. **Lanhe Chu**: Resources; data curation. **Wenchong Tan**: Visualization; investigation. **Cuiping Ye**: Software. **Hangming Dong**: Project administration; supervision; writing—review and editing.

## CONFLICT OF INTEREST STATEMENT

All authors declared that they have no conflict of interest.

## ETHICS STATEMENT

Ethical approval for the research was obtained from the Ethics Committee of Nanfang Hospital of Southern Medical University (No. NFEC‐2023‐398).

## Data Availability

Data will be made available on request.
